# Correlates of physician burnout across regions and specialties: a meta-analysis

**DOI:** 10.1186/1478-4491-11-48

**Published:** 2013-09-28

**Authors:** Raymond T Lee, Bosu Seo, Steven Hladkyj, Brenda L Lovell, Laura Schwartzmann

**Affiliations:** 1 Department of Business Administration, University of Manitoba, 181 Freedman Crescent, Winnipeg, MB R3T 5V4, Canada; 2 Economics Department, University of the Fraser Valley, 33844 King Road, Abbotsford BC V2S 7M7, Canada; 3 Department of Psychology, University of Manitoba, 190 Dysart Road, Winnipeg MB R3T 5V4, Canada; 4 1 Glengarry Drive, Winnipeg MB R3T 2J5, Canada; 5 Facultad de Medicina, Universidad de la República Oriental del Uruguay, Gral Flores 2125 Sede Centrale, CP 11100, Montevideo, Uruguay

**Keywords:** Physician burnout, Work engagement, Health and safety, Mental and physical well-being, Coping strategies, Health behaviors

## Abstract

**Background:**

Health care organizations globally realize the need to address physician burnout due to its close linkages with quality of care, retention and migration. The many functions of health human resources include identifying and managing burnout risk factors for health professionals, while also promoting effective coping. Our study of physician burnout aims to show: (1) which correlates are most strongly associated with emotional exhaustion (EE) and depersonalization (DP), and (2) whether the associations vary across regions and specialties.

**Methods:**

Meta-analysis allowed us to examine a diverse range of correlates. Our search yielded 65 samples of physicians from various regions and specialties.

**Results:**

EE was negatively associated with autonomy, positive work attitudes, and quality and safety culture. It was positively associated with workload, constraining organizational structure, incivility/conflicts/violence, low quality and safety standards, negative work attitudes, work-life conflict, and contributors to poor mental health. We found a similar but weaker pattern of associations for DP.

Physicians in the Americas experienced lower EE levels than physicians in Europe when quality and safety culture and career development opportunities were both strong, and when they used problem-focused coping. The former experienced higher EE levels when work-life conflict was strong and they used ineffective coping. Physicians in Europe experienced lower EE levels than physicians in the Americas with positive work attitudes. We found a similar but weaker pattern of associations for DP.

Outpatient specialties experienced higher EE levels than inpatient specialties when organization structures were constraining and contributors to poor mental health were present. The former experienced lower EE levels when autonomy was present. Inpatient specialties experienced lower EE levels than outpatient specialties with positive work attitudes. As above, we found a similar but weaker pattern of associations for DP.

**Conclusions:**

Although we could not infer causality, our findings suggest: (1) that EE represents the core burnout dimension; (2) that certain individual and organizational-level correlates are associated with reduced physician burnout; (3) the benefits of directing resources where they are most needed to physicians of different regions and specialties; and (4) a call for research to link physician burnout with performance.

## Background

Health care organizations globally realize the need to address physician burnout due to its close linkages with quality of care, retention and migration. A 2008 World Health Organization (WHO) report found that the major factors for turnover and migration were poor or dangerous working conditions, insufficient resources, limited career opportunities, and economic instability [1]. The field of health human resources (HHR) deals with human resource issues for workers in the health sector, and has been suggested as a way to strengthen health system performance and to improve well-being for health professionals [2]. The many functions of HHR include identifying and managing the individual and environmental burnout risk factors, while simultaneously promoting effective coping [3-5].

Burnout is a specific pattern of response to chronic work-related stress that is a serious issue for many physicians [6]. Physician burnout is characterized primarily by a depletion of mental energy, known as emotional exhaustion (EE). With such depletion, providers feel unable to give of themselves, which leads to cynical attitudes and detached feelings toward patients, known as depersonalization (DP). The third burnout dimension is negative self-appraisal, especially in the competencies required to work with others, known as diminished personal accomplishment [6]. Our study will focus on the EE and DP dimensions only.

The frameworks to explain the development of burnout in health professionals have ranged from personal characteristics to work organization variables or a combination of the two. For example, Wiskow *et al*.’s model emphasizes the impact of the work environment, which is influenced by: (a) organizational functionality; (b) organizational culture; (c) management and patient support; (d) staff development; and (e) work-family balance [7]. These elements have been linked to burnout, medical errors and quality of care [7,8]. In turn, burnout is posited to be a risk factor for increased turnover and migration in physicians [2,8,9]. Existing evidence supports models with personal and work characteristics. The three levels of change to reduce burnout risk are: (1) modifying the organizational structure and work processes; (2) improving the fit between the organization and the individual physician, including professional development programs to facilitate better adaption to the work environment; and (3) individual-level actions to reduce stress and poor health symptoms through effective coping and promoting healthy behaviors [2,3,10].

The aims of our study of physicians are to determine which correlates would be most strongly associated with EE and DP, and whether the associations would vary across geographical regions and specialties. The three levels of burnout risk served as the framework for the categorization of variables that we created in this study. Our findings will help the field of HHR to identify personal and work characteristics that are the most significant risk factors for EE and DP, and direct resources most needed to physicians of different regions and specialties.

## Methods

We chose meta-analysis in this study. The use of multi-sample data of physicians from different regions and specialties allows for the examination of a more diverse range of risk factors than would be possible with any single-sample data. Our study followed the Preferred Reporting Items for Systematic Reviews and Meta-Analyses (PRISMA) guidelines and reporting standards [11].

### Literature search

We searched for published studies from 1991 to 2011, using the terms, ‘physician/doctor emotional exhaustion,’ ‘physician/doctor burnout,’ and ‘physician/doctor coping,’ with the search engines: Cochrane, Embase, TheFreeLibrary.com, Google, Google Scholar, LILACS, PsycINFO, PubMed, SciELO and Scopus. Our search yielded 92 studies of medical doctors, but 27 were excluded either because they each included physicians with other health professionals (k = 13), or did not report or respond to email requests for the necessary statistics (k = 14). The remaining (K = 65) sampled exclusively physicians and provided either the sample correlations (r) or statistics that could be converted to r. The studies used in our meta-analysis are listed in the Appendix. Four of these were published in Spanish, with the variables and text recorded and translated into English by the fifth author.

### Procedure

For the coding of sample characteristics, we coded each study sample on the method of survey administration, response rate, sample size, gender distribution, mean years of age, mean years in practice, country of the sample and medical specialty distribution.

For the coding of statistics, the internal consistency reliability estimates (Cronbach’s *α*) and the associations between each correlate with EE and DP were recorded.

For conversion to r, 30 studies provided either a 2 × 2 *χ*^2^, t-ratio, one-way F-ratio or odds ratio (OR). To convert the χ^2^ to r, we used the formula [12]:

(1) $$r={\left(\chi^{2}/N\right)}^{1/2},$$

and to convert the t-ratio or F-ratio to r, we used the formula [12]:

(2) $$r={\left(t^{2}/\left[t^{2}+\mathrm{df}\right]\right)}^{1/2}.$$

To convert the OR to r, we used the formula [13]:

(3) $$r=\left({\mathrm{OR}}^{3/4}-1\right)/\left({\mathrm{OR}}^{3/4}+1\right).$$

For the correlates, the classification of variables as either environmental drivers or constraints were informed by Lewin’s field theory [14], which posits that behavior is the function of the person and the environment, and Lowe and Chan’s classification of healthy work environment indicators [15]. The remaining variables were categorized either as work-life conflict, contributors to good health, contributors to poor mental health or coping strategies (see the correlates under each of the categories in Table 1). The fourth author classified all the variables, and the first author checked the categorizations. The inter-rater agreement for the classification was 100%.

**Table 1:** Weighted mean reliability estimates of all variables

	**k**	**n**	***α***_**correlate**_	***α***_**EE**_	***α***_**DP**_
**Work engagement drivers**					
Recognition/feedback	4	2,125	0.79	0.86	nd
Autonomy	7	2,821	0.71	0.89	0.73
Organization/peer support	3	1,626	nd	0.87	0.69
Adequate resources	2	1,089	nd	0.87	0.68
**Work engagement constraints**					
Professional values	4	686	0.80	nd	0.70
Organization structures	5	1,084	nd	0.88	nd
Inadequate resources	5	1,023	nd	0.89	0.78
Role ambiguity/conflict	3	622	nd	0.87	0.80
Insufficient input	4	437	0.89	0.89	0.79
Workload	19	6,205	0.74	0.88	0.75
Inadequate skills/preparation	4	1,242	nd	0.87	0.80
Position-specific demands	10	3,550	0.76	0.88	0.76
**Work attitudes drivers**	17	13,271	0.61	0.84	0.75
**Work attitudes constraints**	13	4,652	0.74	0.90	0.76
**Health and safety drivers**					
Quality and safety culture	8	8,618	0.67	0.87	0.69
Clinical skills	3	1,033	0.81	0.88	0.69
Professional development	6	1,377	nd	nd	nd
**Health and safety constraints**					
Incivility/conflicts/violence	7	1,816	0.84	0.89	0.79
Lack of quality and safety	15	5,612	0.80	0.88	0.76
**Work**-**life**/**home conflict**	14	3,846	0.80	0.88	0.73
**Contributions to poor mental health**	23	7,345	0.78	0.87	0.69
**Contributions to good health**	6	3,684	nd	0.87	0.80
**Adaptive coping**					
Social support	6	2,297	0.74	0.87	0.80
Problem-focused	8	1,856	0.75	0.87	0.78
**Ineffective coping**	9	2,007	0.78	0.87	0.76

*α*, Cronbach’s *α* weighted mean reliability estimate; DP, depersonalization; EE, emotional exhaustion; k, number of samples; n, sample size across k; nd, no estimate computed.

The correlates classified under work engagement drivers are: recognition/feedback, autonomy, organization/peer support and adequate resources. The correlates classified under work engagement constraints are: professional values (for example, compromise of beliefs), organization structures (for example, supervision, inflexible work arrangements), inadequate resources, role ambiguity/conflict, insufficient input, workload, inadequate skills/preparation, and position-specific demands (for example, patient suffering and emotions). Work attitudes drivers include job and professional satisfaction, and organizational commitment. Work attitudes constraints include lack of motivation, career regret and intent to leave profession. The correlates classified under health and safety drivers are: quality and safety culture (for example, time for patients, management of patient-load), clinical skills, and professional development. The correlates classified under health and safety constraints are: incivility/conflicts/violence, and lack of quality and safety (for example, ergonomics and work-related hazards). Work-life/home conflict is incompatibility between professional and personal obligations and commitments. Contributors to poor mental health include fatigue, anxiety and depression. Contributors to good health include relaxation, hobbies, time for self and others. The correlates classified under adaptive coping are: social support (family, relatives, friends, outside acquaintances) and problem-focused (for example, prioritization of goals, finding meaning, spirituality). Ineffective coping includes over-eating, inactivity and emotion-focused.

### Analyses

For point estimates, our meta-analysis did not include any correlate examined in only one sample. Where a study had two or more separate item measures for a given correlate, we first calculated their mean r with the burnout dimension. For each correlate with a k ≥2, we calculated the weighted mean meta-correlation (*ρ*), and *ρ* corrected for within-sample measurement unreliability (*ρ*_c_), using the formula [16]:

(4) $$\rho_{c}=r_{\mathrm{xy}}/{\left(\alpha_{x}\alpha_{y}\right)}^{1/2},$$

where r_xy_ = r of correlate with burnout dimension, *α*_x_ = reliability estimate of correlate, *α*_y_ = reliability estimate of burnout dimension. We substituted the value of the weighted mean of Cronbach’s *α* or 1 when no reliability estimate was provided.

We considered *ρ*_c_ ≥0.30 to have practical significance for evaluation purposes. For example, a *ρ*_c_ = 0.30 between a work constraint and burnout could mean that 66% of physicians in restrictive environments have high EE levels, and 66% of those in supportive environments have low EE levels [17].

For dispersion around *ρ*_c_, we calculated the variance of *ρ*_c_ (*σ*^2^*ρ*_c_), and the Q-test for homogeneity of r [16], where significance indicates that the associations vary across k. For homogeneous k, the standard error of *ρ*_c_ (SE *ρ*_c_) formula is [18]:

(5) $$\mathrm{SE}\;\rho_{c\mathrm{homogeneous}}=\left(1-\rho_{c}{}^{2}\right)/{\left(n–k\right)}^{1/2},$$

and for heterogeneous k, the SE *ρ*_c_ formula is [18]:

(6) $$\mathrm{SE}\;\rho_{c\mathrm{heterogeneous}}=\left\{\left[\left(1-\rho_{c}{}^{2}\right)/{\left(n–k\right)}^{1/2}\right]+\left(\sigma^{2}{}_{\mathrm{res}}/k\right)\right\},$$

where *σ*^2^_res_ = *σ*^2^*ρ*_c_ – *σ*^2^*ρ*. The SE *ρ*_c_ was used to construct the 95% confidence interval (CI) of *ρ*_c_.

For group differences, for heterogeneous k, we compared the difference in *ρ*_c_’s between regions and between specialty groups using the formula [12]:

(7) $$|Z|-\mathrm{difference}=z_{c1}{}^{’}-z_{c2}{}^{’}/{\left(1/\left[n_{1}–3\right]+1/\left[n_{2}–3\right]\right)}^{1/2},$$

$${\mathrm{wherez}}_{c}{}^{’}=\left(1/2\right)\log_{e}\left(\left[1+\rho_{c}\right]/\left[1-\rho_{c}\right]\right).$$

To check for publication bias, the file drawer problem exists when studies with significant results are published, while those with non-significant results are not reported. This and other types of publication bias are evident when the funnel plot (r by n) is asymmetrical or skewed [19]. Publication bias was checked by: (1) estimating the k with non-significant r that would be needed to increase the *ρ*_c_’s significance level to ≥0.05 (that is, fail-safe k or k_fs_) for each correlate [20], and (2) examining the funnel plots of correlates with k ≥15.

### Analytical software

We used the META 5.3 meta-analysis program (National Collegiate Software Clearinghouse, Raleigh, NC, USA) [21] to estimate the weighted mean of Cronbach’s *α*, *ρ*, *ρ*_c_, *σ*^2^*ρ*_c_, SE *ρ*_c_, 95% CI of *ρ*_c_, Q-test for homogeneity of r, and k_fs_. We used the Microsoft Excel 2010 (Microsoft, Redmond, WA, USA) spreadsheet to convert the *χ*^2^, t-ratio, one-way F-ratio and OR statistics to r’s; compute the K and N, and each group k and n descriptive statistics; and one-way F- and Z-difference tests. We used the Excel scatter chart program to create the funnel plot.

## Results

### Sample characteristics

Table 2 shows that the overall K = 65; N = 28,882; weighted mean for years of age = 45, weighted mean for years in practice = 15, and weighted mean for proportion of males = 73%. All research participants were administered questionnaires either through postal mail (61%), email (4%), in person (24%) or unspecified (12%), and the weighted mean response rate = 62%.

**Table 2:** Sample distribution

	**k**	**n**_**k**_	**Years of age**	**Years in practice**	**Males (%)**	**Response rate (%)**
**Region**						
Americas	26	12,457	42 (14)	16 (3)	74 (13)	53 (24)
Europe	28	13,085	47 (4)	15 (3)	72 (14)	69 (10)
Asia/Australia	11	3,340	41 (4)	15 (3)	76 (12)	68 (14)
F-ratio, df = 2, 62	-	-	2.57^a^	0.90	0.39	6.28^b^
**Specialty group**						
Inpatient	25	10,935	42 (14)	16 (2)	73 (16)	63 (19)
Outpatient	17	4,775	44 (3)	13 (3)	76 (14)	76 (14)
Mixed	23	13,172	47 (4)	15 (3)	73 (12)	57 (18)
F-ratio, df – 2, 62	-	-	1.80	6.49^b^	0.28	5.89^b^
**N**	65	28,882	45 (9)	15 (3)	73 (14)	62 (19)

Values presented as weighted mean (SD). k, number of samples; n_k_, cumulative n across k. ^a^*P* <0.05; ^b^*P* <0.01.

For the Americas, k = 26, n = 12,457; for Europe, k = 28, n = 13,085, and for Australia/Asia, k = 11, n = 3,340. On average, the European samples were older (47 years) than either the American (42 years) or Australian/Asian (41 years) samples. On average, the American samples provided a lower response rate (53%) than either the European (69%) or Australian/Asian (68%) samples.

We divided the samples into three specialty groups. The first was where, within a study sample, all the physicians saw their patients in hospital settings (inpatient specialties); the second was where, within a study sample, all of the physicians saw their patients in non-hospital settings, such as in walk-in clinics (outpatient specialties); and the third was where, within a study sample, some physicians saw patients in hospital settings and other physicians saw patients in non-hospital settings (mix of inpatient and outpatient specialties). For the inpatient specialty group (anesthesiology, internal, gynecology, oncology, otolaryngology, pediatric, surgical), k = 25, n = 10,935; for the outpatient specialty group (emergency medicine, infectious diseases, general/family, ophthalmology, psychiatry), k = 17, n = 4,775; and for the mixed group, k = 23, n = 13,172. On average, the outpatient specialty group had fewer years of practice experience (13 years) than either the inpatient specialty (16 years) or mixed (15 years) groups. On average, the outpatient specialty group provided a higher response rate (76%) than either the inpatient specialty (63%) or mixed groups (57%).

### Reliability estimates

Table 1 shows the k, n and the weighted mean of Cronbach’s *α* of each variable. The weighted mean of Cronbach’s *α* ranged from 0.61 to 0.89 for the correlates, with 15/17 (88%) above 0.70. The weighted mean of Cronbach’s *α* ranged from 0.84 to 0.90 for EE and from 0.68 to 0.80 for DP.

### Overall associations

Tables 3 and 4 show the k, n, *ρ*, *ρ*_c_, *σ*^2^_*ρ*c_, 95% CI of *ρ*_c_, Q-test, and k_fs_. Table 3 reveals that EE had 25 correlates with k ≥2, and 17/25 (68%) had *ρ*_c_’s ≥0.30. Autonomy (*ρ*_c_ = −0.36) was the strongest correlate of the work engagement drivers; workload (*ρ*_c_ = 0.66) and organizational structure (*ρ*_c_ = 0.45) were the strongest correlates of the work engagement constraints. EE was associated with the work attitude drivers (*ρ*_c_ = −0.47) and work attitude constraints (*ρ*_c_ = 0.46). Quality and safety culture (*ρ*_c_ = −0.34) was the strongest correlate of the health and safety drivers; incivility/conflicts/violence (*ρ*_c_ = 0.41), and lack of quality and safety (*ρ*_c_ = 0.42) were equally strong correlates of the health and safety constraints. EE was strongly associated with work-life conflict (*ρ*_c_ = 0.49), and contributors to poor mental health (*ρ*_c_ = 0.62), moderately associated with contributors to good health (*ρ*_c_ = −0.32) and ineffective coping strategies (*ρ*_c_ = 0.33).

**Table 3:** **Meta**-**correlations with emotional exhaustion (EE)**

**Correlates**	**k**	**n**	**ρ**	**ρ**_**c**_	**σ**^**2**^_**ρc**_	**95% CI ρ**_**c**_	**Q**	**k**_**fs**_
**Work Engagement Drivers**								
Recognition/feedback	4	2,125	−0.17	−0.20^b^	0.003	−0.24 to −0.11	6.69	10
Autonomy	6	2,189	−0.26	**−0.36**^b^	0.021	−0.60 to −0.01	53.09^b^	30
Organization/peer support	4	2,748	−0.15	−0.18^b^	0.002	−0.21 to −0.09	4.18	8
Adequate resources	2	1,089	−0.14	−0.15^b^	0.002	−0.18 to −0.11	2.35	4
**Work Engagement Constraints**								
Professional values	2	91	0.36	**0.42**^b^	0.000	0.41 to 0.42	0.01	13
Organization structures	5	1,084	0.44	**0.45**^b^	0.038	0.08 to 0.82	63.41^b^	40
Inadequate resources	5	1,023	0.34	**0.36**^b^	0.012	0.18 to 0.53	15.16^b^	31
Role ambiguity/conflict	3	622	0.23	0.24^b^	0.007	0.12 to 0.34	5.14	11
Insufficient input	4	437	0.34	**0.36**^b^	0.017	0.15 to 0.56	9.79^a^	23
Workload	19	6,205	0.51	**0.66**^b^	0.018	0.28 to 0.79	206.09^b^	183
Inadequate skills/preparation	4	1,242	0.24	0.26^b^	0.002	0.25 to 0.27	3.34	16
Position specific demands	10	3,550	0.32	**0.40**^b^	0.004	0.27 to 0.43	18.19^a^	61
**Work Attitude Drivers**	16	12,323	−0.30	**−0.47**^b^	0.022	−0.61 to −0.02	328.50^b^	85
**Work Attitude Constraints**	13	4,652	0.35	**0.46**^b^	0.008	0.21 to 0.52	50.15^b^	82
**Health & Safety Drivers**								
Quality & safety culture	7	8,226	−0.23	**−0.34**^b^	0.016	−0.50 to 0.00	143.72^b^	28
Clinical Skills	3	1,033	−0.08	−0.08^a^	0.004	−0.16 to −0.00	4.39	2
Professional development	6	1,377	−0.31	**−0.31**^b^	0.014	−0.51 to −0.10	23.74^b^	31
**Health & Safety Constraints**								
Incivility/conflicts/violence	7	1,816	0.34	**0.41**^b^	0.015	0.14 to 0.58	34.61^b^	43
Lack of quality & safety	15	5,612	0.34	**0.42**^b^	0.007	0.22 to 0.51	50.11^b^	94
**Work–Life/Home Conflict**	13	3,817	0.40	**0.49**^b^	0.019	0.16 to 0.69	102.49^b^	98
**Contributors to Poor Mental Health**	23	7,345	0.49	**0.62**^b^	0.013	0.30 to 0.71	168.99^b^	210
**Contributors to Good Health**	6	4,806	−0.28	**−0.32**^b^	0.01	−0.48 to −0.10	57.76^b^	29
**Adaptive Coping**								
Social support	7	3,448	−0.17	−0.26^b^	0.007	−0.32 to −0.04	28.34^b^	18
Problem-focused	9	3,007	−0.20	−0.29^b^	0.009	−0.38 to −0.04	30.42^b^	29
**Ineffective Coping**	9	3,129	0.22	**0.33**^b^	0.009	0.07 to 0.50	31.28^b^	33

95% CI *ρ*_c_, confidence interval; *σ*^*2*^_*ρ*c_, variance of *ρ*_c_; k, number of samples; k_fs_, fail-safe k for critical *ρ*_c_ ≥0.05; n, sample size across k; *ρ*, weighted mean meta-correlation; *ρ*_c_, *ρ* after correcting for measurement unreliability with value ≥0.30 in bold; Q, homogeneity of r test, where significance indicates that the *ρ*_c_’s vary across k. ^a^*P* <0.05; ^b^*P* <0.001.

**Table 4:** **Meta**-**correlations with depersonalization (DP)**

**Correlates**	**k**	**n**	**ρ**	**ρ**_**c**_	**σ**^***2***^_**ρc**_	**95% CI ρ**_**c**_	**Q**	**k**_**fs**_
**Work Engagement Drivers**								
Recognition, feedback	3	853	−0.04	−0.05	0.004	−0.07 to −0.01	3.25	<1
Autonomy	5	1,769	-0.17	−0.24^b^	0.003	−0.42 to 0.01	22.36^b^	15
Organization/peer support	3	1,597	−0.08	−0.09^b^	0.004	−0.18 to 0.01	6.17^a^	2
**Work Engagement Constraints**								
Professional values	4	686	0.28	**0.36**^b^	0.003	0.34 to 0.37	2.23	21
Organization structures	2	198	0.47	**0.47**^b^	0.003	0.46 to 0.47	1.02	17
Role Ambiguity/conflict	2	593	0.23	0.26^b^	0.003	0.25 to 0.26	0.05	8
Workload	12	3,899	0.26	0.29^b^	0.011	0.11 to 0.48	48.80^b^	58
Inadequate skills/preparation	3	679	0.28	**0.35**^b^	0.001	0.34 to 0.36	0.53	15
Position specific demands	7	1,773	0.28	**0.38**^b^	0.005	0.27 to 0.41	10.30^b^	41
**Work Attitudes Drivers**	13	11,206	−0.24	**−0.36**^b^	0.008	**−**0.43 to **−**0.10	100.07^b^	55
**Work Attitudes Constraints**	7	1,945	0.24	**0.32**^b^	0.009	0.11 to 0.40	19.44^b^	33
**Health & Safety Drivers**								
Quality & safety culture	7	7,640	−0.24	**−0.35**^b^	0.006	−0.41 to −0.12	51.09^b^	30
Clinical skills	2	975	−0.11	**−**0.15^b^	0.001	−0.15 to −0.16	1.12	4
Professional development	5	1,348	−0.18	−0.18^b^	0.000	−0.18 to −0.18	0.61	13
**Health & Safety Constraints**								
Incivility/conflicts/violence	3	220	0.42	**0.51**^b^	0.01	0.49 to 0.54	3.18	25
Lack of quality & safety	8	2,573	0.27	**0.33**^b^	0.012	0.10 to 0.53	36.49^b^	42
**Work–Life/ Home Conflict**	9	2,511	0.27	**0.34**^b^	0.01	0.13 to 0.47	29.06^b^	39
**Contributors to Poor Mental Health**	17	5,411	0.27	**0.34**^b^	0.008	0.14 to 0.42	50.96^b^	78
**Contributors to Good Health**	5	3,387	−0.15	−0.15^b^	−0.16	−0.27 to −0.04	16.71^b^	11
**Adaptive Coping**								
Social support	5	1,866	−0.16	−0.21^b^	0.004	−0.23 to −0.10	7.10	11
Problem-focused	7	1,647	−0.14	−0.18^b^	0.004	−0.16 to −0.14	7.31	14
**Ineffective Coping**	9	2,007	0.19	0.24^b^	0.012	0.02 to 0.40	26.13^b^	25

95% CI *ρ*_c_, confidence interval; *σ*^*2*^_*ρ*c_, variance of *ρ*_c_; k, number of samples; k_fs_, fail-safe k for critical *ρ*_c_ ≥0.05; n, sample size across k; *ρ*, weighted mean meta-correlation; *ρ*_c_, *ρ* after correcting for measurement unreliability with value ≥0.30 in bold; Q, homogeneity of r test, where significance indicates that the *ρ*_c_’s vary across k. ^a^*P* <0.05; ^b^*P* <0.001.

**Figure 1:**
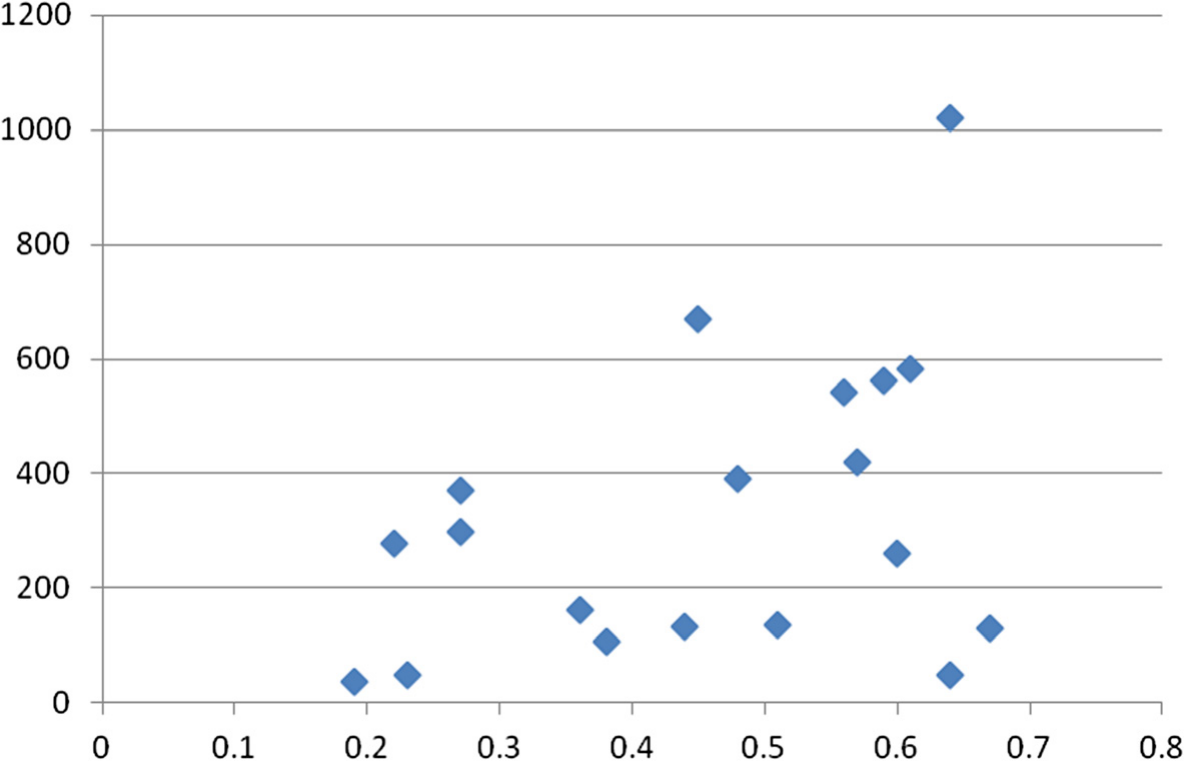
**Funnel plot of workload with emotional exhaustion (EE).** x-axis: r; y-axis: n. k = 19; range of n: 37 to 1,021.

**Figure 2:**
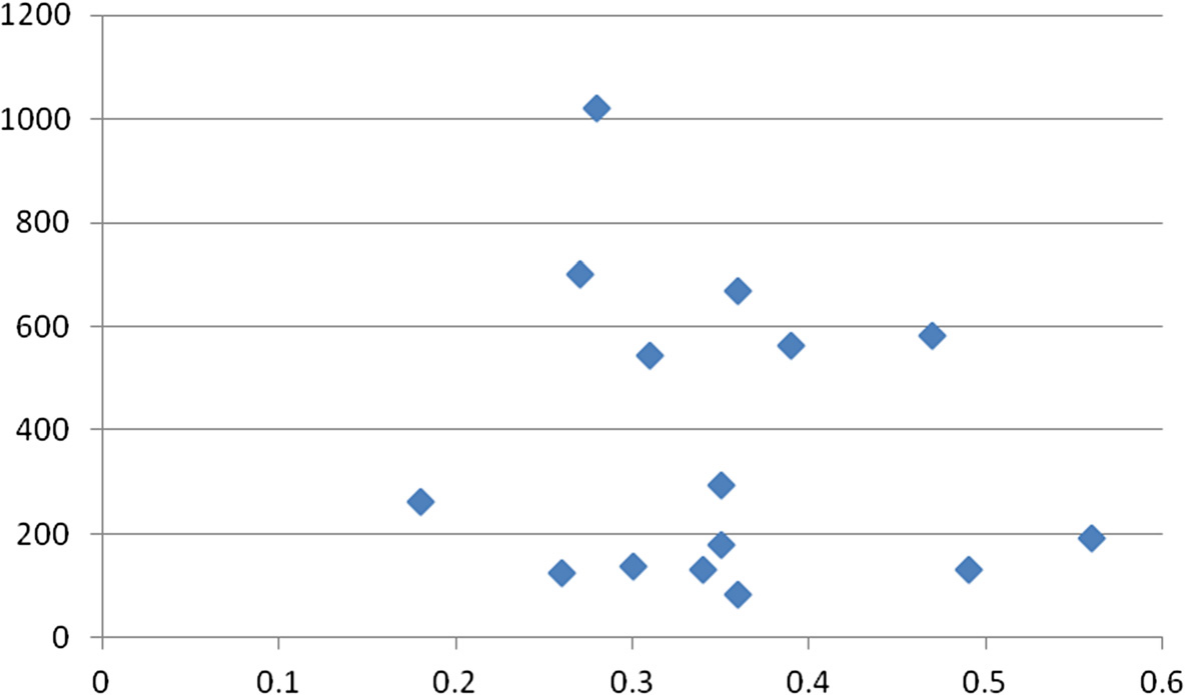
**Funnel plot of lack of quality and safety with emotional exhaustion (EE).** x-axis: r; y-axis: n. k = 15; range of n: 84 to 1,021.

**Figure 3:**
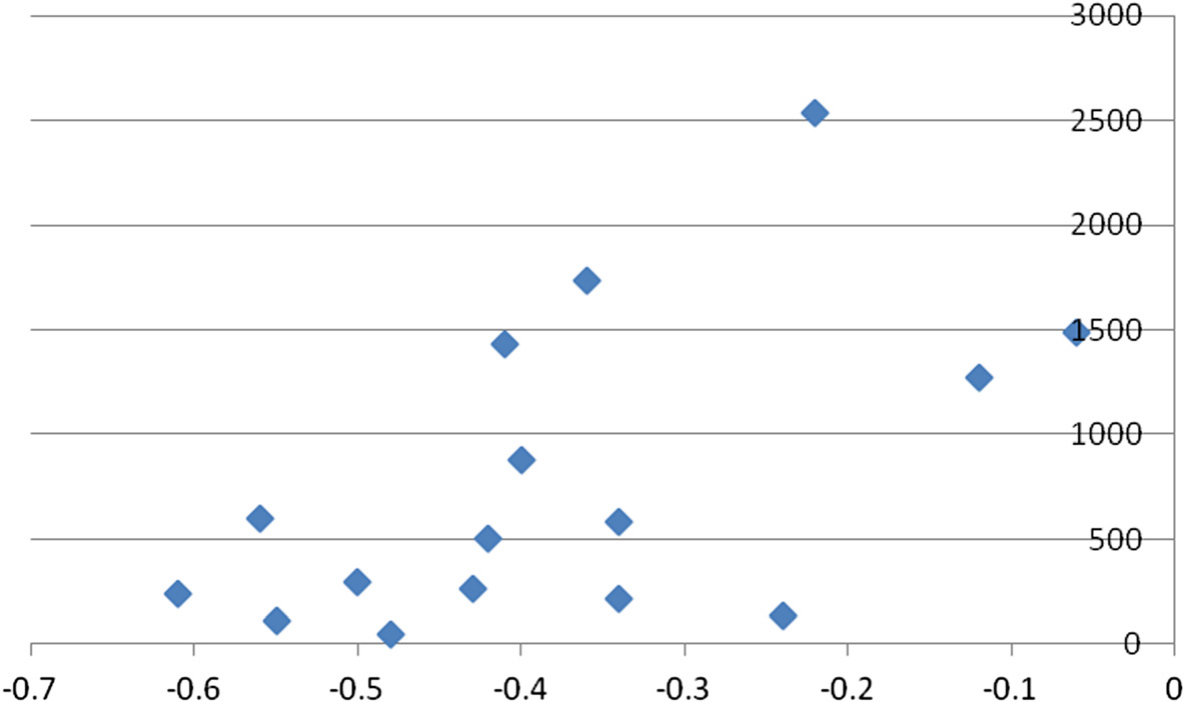
**Funnel plot of work attitudes drivers with emotional exhaustion (EE).** x-axis: r; y-axis: n. k = 16; range of n: 50 to 2,536.

**Figure 4:**
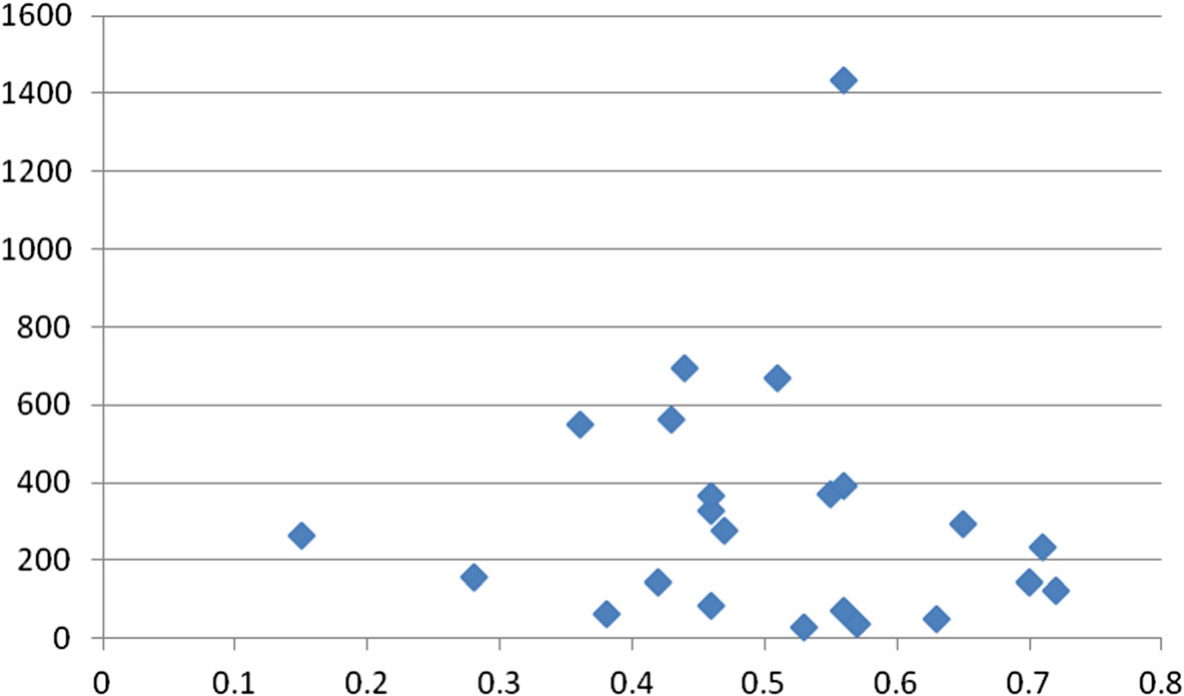
**Funnel plot of contributors to poor mental health with emotional exhaustion (EE).** x-axis: r; y-axis: n. k = 23, range of n: 29 to 1,435.

EE had 22/25 (88%) correlates with k_fs_ ≥10, and 17/25 (68%) correlates with a k_fs_/k ratio ≥4/1, indicating minimal risks of the file drawer problem. Figures 1, 2, 3 and 4 show the plots of r by n for workload, work attitude drivers, lack of quality and safety, and contributors to poor mental health. All four were funnel-shaped with three symmetrical, indicating minimal risks of publication bias [17].

Table 4 reveals that DP had 22 correlates with k ≥2, and 11/22 (50%) had *ρ*_c_’s ≥0.30. Three correlates, adequate resources, inadequate resources and insufficient inputs, each had k = 1 and were not included in the meta-analysis for DP. Organizational structure (*ρ*_c_ = 0.47) was the strongest correlate of the work engagement constraints. DP was moderately associated with the work attitude drivers (*ρ*_c_ = −0.36) and work attitude constraints (*ρ*_c_ = 0.32). Quality and safety culture (*ρ*_c_ = −0.35) was the strongest correlate of the health and safety drivers; incivility/conflicts/violence (*ρ*_c_ = 0.51) was a stronger correlate than lack of quality and safety (*ρ*_c_ = 0.33) of the health and safety constraints. DP was moderately associated with work-life conflict (*ρ*_c_ = 0.34), and contributors to poor mental health (*ρ*_c_ = 0.34).

**Figure 5:**
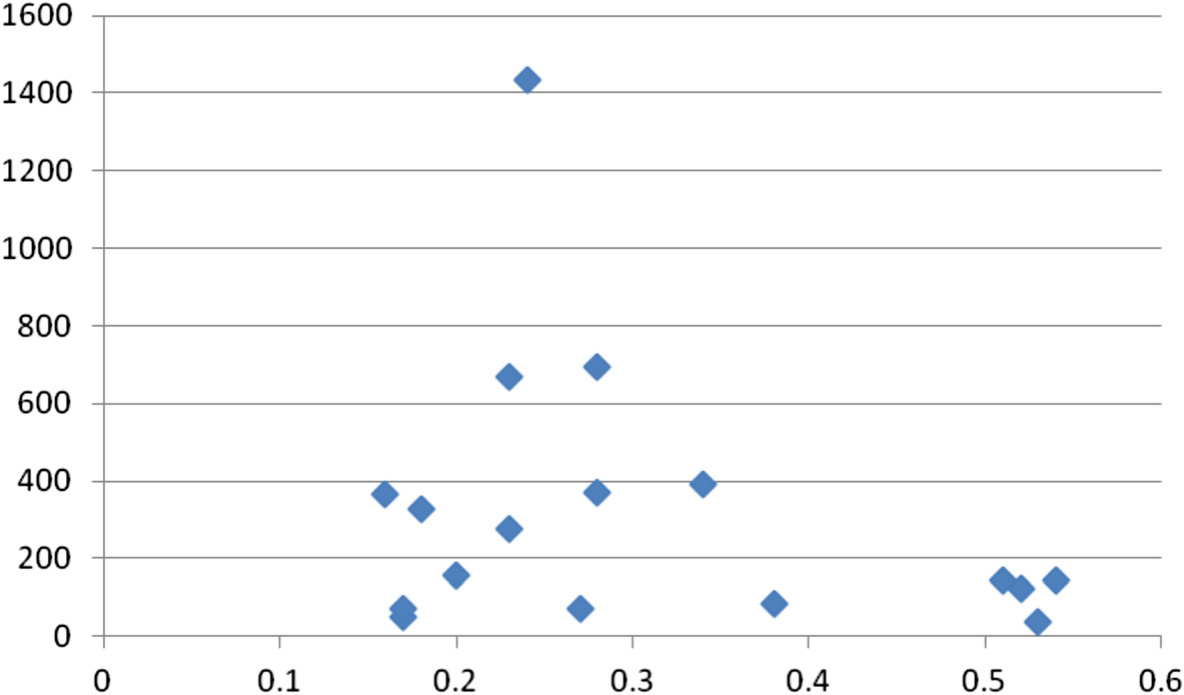
**Funnel plot of contributors to poor mental health with depersonalization (DP).** x-axis: r; y-axis: n. k = 17; range of n: 37 to 1,435.

DP had 18/22 (82%) correlates with k_fs_ ≥10, and 13/22 (59%) correlates with a k_fs_/k ratio ≥4/1, indicating minimal risks of the file drawer problem. Figure 5 shows the plot of r by n for contributors to poor mental health. It was funnel-shaped and symmetrical, indicating a minimal risk of publication bias.

### Group differences

The interpretation of the overall *ρ*_c_’s must be qualified due to heterogeneity of r’s across k on both burnout dimensions. The r’s were heterogeneous on 18/25 (72%) correlates for EE, and on 12/22 (55%) correlates for DP. For these correlates, we compared the significance of *ρ*_c_ differences between the two largest regions, the Americas and Europe, and between inpatient and outpatient specialty groups. We did not compare with the mixed group because both inpatient and outpatient specialties were combined in a given sample.

Tables 5 and 6 show the k, n, *ρ*_c_ and Z-difference between regions. Table 5 reveals that EE had 13/18 (72%) correlates with significant *ρ*_c_ differences. The *ρ*_c_’s of the Americas were stronger than Europe on 11 correlates. The most notable differences were in quality and safety culture (−0.56 versus −0.25), professional development (−0.41 versus −0.22), work-life conflict (0.57 versus 0.40), problem-focused coping (−0.44 versus −0.17), and ineffective coping (0.53 versus 0.32). Physicians in the Americas were at lower risk than physicians in Europe for EE when quality and safety culture and career development opportunities were present, and problem-focused coping was used. The former were at higher risk than the latter when work-life conflict was present, and ineffective coping was used. The *ρ*_c_ of the work attitude drivers was stronger for Europe (−0.64) than for the Americas (−0.28), indicating that the former was at lower risk than the latter for EE when their attitudes were positive.

**Table 5:** Regional differences for emotional exhaustion (EE)

	**Americas**			**Europe**			
**Correlates**	**k**	**n**	***ρ***_**c**_	**k**	**n**	***ρ***_**c**_	**Z**-**dif**
**Work engagement drivers**							
Autonomy	2	860	−**0.49**	3	1,279	−0.24	6.39^b^
**Work engagement constraints**							
Organization structures	1	62	0.48	2	857	0.44	0.42
Inadequate resources	3	431	0.33	1	563	0.37	0.78
Insufficient input	2	324	0.37	1	84	0.25	1.11
Workload	9	2,746	**0.70**	8	2,876	0.63	4.79^b^
Position-specific demands	3	578	**0.46**	6	2,429	0.37	2.13^a^
**Work attitudes drivers**	6	6,114	−0.28	7	5,705	**−0.64**	25.42^b^
**Work attitudes constraints**	6	1,474	**0.55**	4	1,934	0.38	6.36^b^
**Health and safety drivers**							
Quality and safety culture	2	2,714	−**0.56**	4	4,815	−0.25	15.74^b^
Professional development	2	652	−**0.41**	2	646	−0.22	3.76^b^
**Health and safety constraints**							
Incivility/conflicts/violence	3	431	0.34	3	1,356	0.43	1.89
Lack of quality and safety	6	1,921	**0.47**	8	3,148	0.39	3.42^b^
**Work**-**life**/**home conflict**	7	1,881	**0.57**	5	1,907	0.40	7.04^b^
**Contributors to poor mental health**	5	1,157	0.57	13	5,018	**0.65**	3.86^b^
**Contributors to good health**	2	879	−0.33	2	2,233	0.30	0.60
**Adaptive coping**							
Social support	4	1,253	−**0.35**	1	501	−0.22	2.68^a^
Problem-focused	2	342	−**0.44**	3	892	−0.17	4.81^b^
**Ineffective coping**	1	133	**0.53**	7	1,845	0.32	2.36^a^

k, number of samples; n, sample size across k; *ρ*_c_, weighted mean meta-correlation after correcting for measurement unreliability; Z-dif, tests whether *ρ*_c_’s are significantly different from each other. Bold values indicate the stronger of the two *ρ*_c_’s for the correlate. ^a^*P* <0.05; ^b^*P* <0.001, two-tailed.

**Table 6:** Regional differences for depersonalization (DP)

		**Americas**			**Europe**		
**Correlates**	**k**	**n**	***ρ***_**c**_	**k**	**n**	***ρ***_**c**_	**Z**-**dif**
**Work engagement drivers**							
Autonomy	2	860	−0.28	2	859	−0.20	1.70
Organization/peer support	1	133	−0.15	1	1,435	−0.08	0.80
**Work engagement constraints**							
Workload	3	993	0.32	7	2,313	0.33	0.29
Position-specific demands	2	385	**0.51**	4	815	0.32	2.71^a^
**Work attitudes drivers**	5	4,842	−0.31	5	5,148	**−0.38**	4.19^b^
**Work attitudes constraints**	3	765	**0.38**	3	913	0.25	2.89^a^
Quality and safety culture	2	2,714	**−0.41**	4	4,229	−0.33	3.86^b^
**Health and safety constraints**							
Lack of quality and safety	2	760	**0.47**	5	1,270	0.29	4.33^b^
**Work**-**life**/**home conflict**	4	1,138	**0.42**	4	1,344	0.27	4.14^b^
**Contributors to poor mental health**	4	608	**0.45**	10	3,898	0.33	3.23^b^
**Contributors to good health**	1	582	**−0.27**	2	2,233	−0.13	3.02^a^
**Ineffective coping**	1	133	0.32	7	1,845	0.23	1.02

k, number of samples; n, sample size across k; *ρ*_c_, weighted mean meta-correlation after correcting for measurement unreliability; Z-dif, tests whether *ρ*_c_’s are significantly different from each other. Bold values indicate the stronger of the two *ρ*_c_’s for the correlate. ^a^*P* <0.05; ^b^*P* <0.001, two-tailed.

Table 6 reveals that DP had 8/12 (67%) correlates with significant *ρ*_c_ differences. The *ρ*_c_’s of the Americas were stronger than Europe on seven correlates. The most notable differences were in lack of quality and safety (0.47 versus 0.29), and work-life conflict (0.42 versus 0.27). Physicians in the Americas were at higher risk than physicians in Europe for DP when quality and safety was compromised and work-life conflict was present.

Tables 7 and 8 show the k, n, *ρ*_c_ and Z-difference between specialty groups. Table 7 reveals that EE had 8/16 (50%) correlates with significant *ρ*_c_ differences. The *ρ*_c_’s of the outpatient specialties were stronger than the inpatient specialties on seven correlates. The most notable differences were in autonomy (−0.79 versus −0.57), organizational structures (0.72 versus 0.32) and contributors to poor mental health (0.77 versus 0.56). The former were at higher risk than the latter for EE when the organization of work was constraining and poor mental health were present, but were at lower risk for EE with autonomy and use of problem-focused coping. The *ρ*_c_ of the work attitude drivers was stronger for the inpatient specialties (−0.44) than for the outpatient specialties (−0.29), indicating that the former was at lower risk for EE when their attitudes were positive.

**Table 7:** Specialty group differences for emotional exhaustion (EE)

	**Inpatient**			**Outpatient**			
**Correlates**	**k**	**n**	***ρ***_**c**_	**k**	**n**	***ρ***_**c**_	**Z**-**dif**
**Work engagement drivers**							
Autonomy	2	843	−0.57	1	50	**−0.79**	2.79^a^
**Work engagement constraints**							
Organization structures	3	654	0.32	1	294	**0.72**	8.27^b^
Inadequate resources	2	592	0.38	3	431	0.33	0.96
Insufficient input	1	29	0.44	3	408	0.35	0.54
Workload	9	4,366	**0.72**	5	558	0.65	2.82^a^
Position-specific demands	4	2,519	0.38	4	591	0.42	1.00
**Work attitudes drivers**	5	2,854	**−0.44**	5	2,236	−0.29	5.94^b^
**Work attitudes constraints**	6	2,711	0.44	5	1,346	0.44	0.06
**Health and safety drivers**							
Professional development	3	1,112	−0.31	1	145	−0.34	0.35
**Health and safety constraints**							
Incivility/conflicts/violence	2	1,007	0.36	5	809	**0.48**	3.02^a^
Lack of quality and safety	8	4,518	0.41	5	839	**0.50**	3.00^a^
**Work**-**life**/**home conflict**	6	2,055	0.49	3	471	0.52	0.94
**Contributors to poor mental health**	10	3,498	0.56	5	791	**0.77**	9.86^b^
**Adaptive coping**							
Social support	3	1,190	−0.18	1	133	−0.17	1.18
Problem-focused	5	1,543	−0.29	1	133	**−0.48**	2.51^a^
**Ineffective coping**	4	1,469	0.32	2	217	0.47	1.80

k, number of samples; n, sample size across k; *ρ*_c_, weighted mean meta-correlation after correcting for measurement unreliability; Z-dif, tests whether *ρ*_c_’s are significantly different from each other. Bold values indicate the stronger of the two *ρ*_c_’s for the correlate. ^a^*P* <0.05; ^b^*P* <0.001, two-tailed.

**Table 8:** Specialty group differences for depersonalization (DP)

	**Inpatient**			**Outpatient**			
**Correlates**	**k**	**n**	***ρ***_**c**_	**k**	**n**	***ρ***_**c**_	**Z**-**dif**
**Work engagement drivers**							
Autonomy	2	843	−0.33	1	50	−0.51	1.40
Organization/peer support	1	29	−0.50	1	133	−0.15	1.85
**Work engagement constraints**							
Workload	6	2,485	0.37	1	133	0.34	0.46
Position-specific demands	3	583	0.26	1	207	0.43	1.72
**Work attitudes drivers**	2	1,083	**−0.45**	5	2,890	−0.29	4.93^a^
**Work attitudes constraints**	2	1,083	0.28	3	267	0.27	0.22
**Health and safety constraints**							
Lack of quality and safety	5	2,234	0.32	1	84	0.34	0.24
**Work**-**life**/**home conflict**	5	1,402	0.32	2	278	**0.46**	2.52^a^
**Contributors to poor mental health**	5	1,858	0.35	4	497	**0.63**	7.41^b^
**Ineffective coping**	5	1,498	0.22	2	217	0.28	0.78

k, number of samples; n, sample size across k; *ρ*_c_, weighted mean meta-correlation after correcting for measurement unreliability; Z-dif, tests whether *ρ*_c_’s are significantly different from each other. Bold values indicate the stronger of the two *ρ*_c_’s for the correlate. ^a^*P* <0.05; ^b^*P* <0.001, two-tailed.

Table 8 reveals that DP had 3/10 (30%) correlates with significant *ρ*_c_ differences. The *ρ*_c_’s of the outpatient specialties were stronger than the inpatient specialties for work-life/home conflict (0.46 versus 0.32), and contributors to poor mental health (0.63 versus 0.35), indicating that the former were at higher risk than the latter for DP when work-life conflict and poor mental health was present. The *ρ*_c_ of the work attitude drivers was stronger *ρ*_c_ for the inpatient specialties (−0.45) than the outpatient specialties (−0.29), indicating that the former was at lower risk for DP when their attitudes were positive.

## Discussion

**Figure 6:**
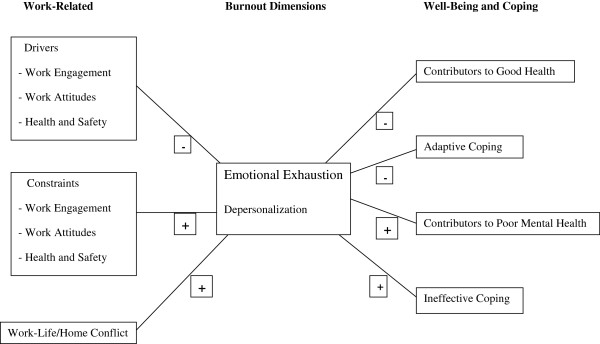
**Summary of overall associations.** The direction of *ρ*_c_ is indicated by either ‘+’ or ‘−’, with the large font indicating high magnitude of association.

Figure 6 shows the overall associations and reveals some significant trends. The *ρ*_c_’s with burnout were stronger for constraints than for drivers. Similarly, the *ρ*_c_’s with burnout were stronger for work-life conflict and contributors to poor mental health than contributors to good health. EE was more strongly associated with a greater number of correlates than DP. EE’s stronger ties with the environmental drivers and constraints support Maslach’s contention that it represents the core aspect of burnout [22]. The results also support Maslach’s position that EE is more closely tied to health states. The implication is that while drivers are important, the management of constraints may be even more critical for physicians who experience high EE. In summary, our findings suggest that attempts to reduce burnout risk could operate at three levels: individual (healthy lifestyle/behaviors, adequate coping), the individual and the environment (social support structures, relationships, improving person-organization fit), and at the organizational level (adequate working conditions, organization of work, design) [7,9,10].

### Drivers and constraints of EE

Excessive and unevenly distributed workloads are fairly pervasive constraints, and were strongly associated with EE. The improvement of work processes, flow and interpersonal relationships (quality and safety) were drivers associated with reduced EE. A positive work attitude was another driver associated with reduced EE, and suggests the benefit of fostering greater organizational commitment and career satisfaction. Contributors to poor mental health and work/life conflict were strongly associated with EE, indicating the importance of self-care practices, and improved personal and family management.

### Drivers and constraints of DP

A culture of quality and safety and positive work attitudes were critical drivers associated with reduced DP. Contributors to poor mental health and work/life conflict were associated with DP, although the link between poor mental health and EE was much stronger. Again, our findings underscore the necessity of self-care and finding the right career balance.

### Americas versus Europe

The moderating role of region may be partly due to age differences, with physicians from Europe, on average, being five years older than physicians from the Americas. The age-related maturity may have enabled many of the European physicians to better manage the risk factors. Work-life conflict had stronger associations with burnout in physicians from the Americas than physicians from Europe. In addition to the greater maturity levels, physicians from Europe may have received more extended family support than their colleagues from the Americas. A caveat worth noting is that the dissimilar mean response rates between the physicians from Europe and the Americas may have distorted the group differences in *ρ*_c_’s.

For physicians from the Americas, possible ways to reduce EE include rebalancing the constraints of heavy workload and position-specific demands, while improving quality and safety culture, and professional development. For physicians from Europe, possible ways to reduce EE include managing the factors that contribute to poor health. For physicians from the Americas, possible ways to reduce DP include cultivating a climate that generates positive work attitudes, quality and safety culture, work-life balance, and managing the contributors to poor health.

Our findings suggest that factors other than culture and economics should be considered when comparing these two regions. The challenges and complexities inherent in the physicians’ work may limit the scope of any proposed changes. Redistributing patient-loads may be difficult within health systems faced with chronic resource constraints. The changes in work routines, resource distribution and decision-making processes may be resisted by physicians and other health professionals. Applying Wiskow *et al*.’s three levels of change require an integrated, systems approach based on careful planning and coordinated implementation [7].

### Inpatient versus outpatient specialties

The moderating role of specialties may be partly due to differences in practice experience, with inpatient specialties having, on average, three more years of practice than outpatient specialties. The increased knowledge commensurate with experience may have enabled many of the inpatient specialties to better manage the risk factors. The stronger associations between EE and its correlates for the outpatient specialties also suggest increased difficulties with work organization and processes due to geographical isolation, and the transient nature of patient relations. The most significant finding was the link between contributors to poor mental health and burnout, with the *ρ*_c_ stronger for outpatient than inpatient specialties. This may indicate that managing the financial, logistical and other business-related needs of outreach clinics exacts a severe toll on their health. Their health deterioration is associated with increased workload and challenges over and above the demands of clinical practice. Outpatient specialties in managed care systems may experience negative health states due to the highly regulated environment, which limits their autonomy, decision input and ability to develop long-term professional relationships with patients [23]. Possible ways to reduce EE for them include providing informational and material resources, training/development programs, and collegial and administrative support. A caveat worth noting is that the dissimilar mean response rates between the outpatient and inpatient specialties may have distorted the group differences in *ρ*_c_’s.

### Study limitations

One study limitation is the lack of uniform standards in the reporting of sample characteristics and r’s. The conversion of ORs may have yielded imprecise r estimates [13]. A second is the inability to infer causality. Did poor health lead to physician burnout, or vice-versa? Similarly, did work-life conflict precede or follow from burnout? A third is that health contexts may have influenced some of our results, but characteristics of health systems (for example, public versus private) were not always reported. A fourth is the paucity of research on physicians in Africa or the Middle East. Finally, we were unable to collect or interpret studies published in languages other than English or Spanish.

## Conclusions

Our study found that reducing the individual and organizational-level risk factors is associated with decreased burnout. Documenting the regional and specialty differences lays the foundation for directing resources where they are most needed. Our findings also reveal the lack of research linking physician burnout with performance. A US study found that physician DP was associated with diminished patient satisfaction and longer post-discharge recovery time [24]. Additional studies could link physician burnout with quality of care and medical errors, which have been found to be negatively associated with patient safety and recovery [25]. Research could examine how physician burnout relates to health behaviors, professional development, communication skills, and overall quality of life.

## Appendix

1. Bovier PA, Arigoni F, Schneider M, Gallacchi MB. Relationships between work satisfaction, emotional exhaustion, and mental health among Swiss primary care physicians.*Eur J Pub Health* 2009; **19:**611–617.

2. Embriaco N, Azoulay E, Barrau K, Kentish N, Pochard F, Loundou A, Papazian L: High level of burnout in intensivists. *Am J Respir Crit Care Med* 2007; **175:**686–692.

3. Kuerer HM, Eberlein TJ, Pollock RE, Huschka M, Baile WF, Morrow M, Michelassi F, Singletary SE, Novotny P, Sloan J, Shanafelt TD: Career satisfaction, practice patterns, and burnout among surgical oncologists: report of the quality of life of members of the Society of Surgical Oncology. *Ann Surg Oncol* 2007; **14:**3043–3053.

4. Campbell DA, Sonnad SS, Eckhauser FE, Campbell KK, Greenfield LJ: Burnout among American surgeons. *Surgery* 2001; **130:**696–705.

5. Kumar S, Fischer J, Robinson E, Hatcher S, Bhagat RN: Burnout and job satisfaction in New Zealand psychiatrists: a national study. *Int J Soc Psychiatry* 2007; **53:**306–316.

6. Korkeila JA, Töyry S, Kumpulainen K, Toivola JM, Räsänen K, Kalimo R: Burnout and self-perceived health among Finnish psychiatrists and child psychiatrists: a national survey. *Scand J Public Health* 2003; **31:**85–91.

7. Visser MRM, Smets EMA, Oort FJ, de Haes HCJM: Stress, satisfaction, and burnout among Dutch medical specialists. *CMAJ* 2003; **168:**271–275.

8. Asai M, Morita T, Akechi T, Sugawara Y, Fujimon M, Akizuki N, Nakano T, Uchitomi Y: Burnout and psychiatric morbidity among physicians engaged in end-of-life care for cancer patients: a cross-sectional nationwide survey in Japan. *Psychooncology* 2006; **16:**421–428.

9. Deckard GJ, Hicks LL, Hamory BH: The occurrence and distribution of burnout among infectious diseases physicians. *J Infect Dis* 1992; **165:**224–228.

10. Bargellini A, Barbieri A, Rovesti S, Vivoli R, Roncaglia R, Borella P: Relation between immune variables and burnout in a sample of physicians. *Occup Environ Med* 2000; **57:**453–457.

11. Dickinson-Bannack ME, González-Salinas C, Fernández-Ortega MA, Palomeque RP, González Quintanilla E, Hernández-Vargas I: Burnout syndrome among Mexican primary care physicians. *Archivos en Medicina Familiar* 2007; **9:**75–79.

12. Winefield HR, Anstey TJ: Job stress in general practice: practitioner age, sex, and attitudes as predictors. *Fam Pract* 1991; **8:**140–144.

13. Morais A, Maia P, Azevedo A, Amaral C, Tavares J: Stress and burnout among Portuguese anaesthesiologists.*Eur J Anaesthesiol* 2006; **23:**433–439.

14. Montgomery AJ, Panagopolou E, Benos A: Work-family interference as a mediator between job demands and job burnout among doctors. *Stress and Health* 2006; **22:**203–212.

15. Grassi L, Magnani K, Ercolani M: Attitudes toward euthanasia and physician-assisted suicide among Italian primary care physicians.*J Pain Symptom Manage* 1999; **17:**188–196.

16. Oyzurt, A. Hayran 0, Sur H: Predictors of burnout and job satisfaction among Turkish physicians. *QJM* 2006; **99:**161–169.

17. AI-Dubai S, Rampal K: Prevalence and associated factors of burnout among doctors in Yemen. *J Occup Health* 2010; **52:**58–65.

18. Lemkau J, Rafferty J, Gordon Jr R: Burnout and career-choice regret among family practice physicians in early practice.* Fam Pract Res J* 1994; **14:**213–222.

19. Lee FJ, Stewart M, Brown JB: Stress, burnout, and strategies for reducing them. *Can Fam Physician* 2008; **54:**234–235.

20. Shanafelt TD, Balch CM, Bechamps G, Russell T, Dyrbye L, Satele D, Collicott P, Novotny PJ, Sloan J, Freischlag J: Burnout and medical errors among American surgeons. *Ann Surg* 2010; **251:**995–1000.

21. Benbow SM, Jolley DJ: Burnout and stress amongst old age psychistrists. *Int J Geriatr Psychiatry* 2002; **17:**710–714.

22. Ádám S, Györffy Z, Susánszky É: Physician burnout in Hungary: a potential role for work family conflict. *J Health Psychol* 2008; **13:**847–856.

23. Shanafelt TD, West CP, Sloan JA, Novotny PJ, Poland GA, Menaker R, Rummans TA, Dyrbye LN: Career fit and burnout among academic faculty. *Arch Intern Med* 2009; **169:**990–995.

24. Sharma A, Sharp DM, Walker LG, Monson JRT: Stress and burnout in colorectal and vascular surgical consultants working in the UK National Health Service.*Psychooncology* 2008; **17:**570–576.

25. Grassi L, Magnani K: Psychiatric morbidity and burnout in the medical profession: an Italian study of general practitioners and hospital physicians. *Psychother Psychosom* 2000; **69:**329–334.

26. Stafford L, Judd F: Mental health and occupational wellbeing of Australian gynaecologic oncologists. *Gynecol Oncol* 2010; **116:**526–532.

27. Bruce SM, Conaglen HM, Conaglen JV: Burnout in physicians: a case for peer-support. *Intern Med J* 2005; **35:**272–278.

28. Wu S, Zhu W, Li H, Wang Z, Wang M: Relationship between job burnout and occupational stress among doctors in China. *Stress and Health* 2008; **24:**143–149.

29. Yost WB, Eshelman A, Raoufi M, Abouljoud MS: A national study of burnout among American transplant surgeons. *Transplant Proc* 2005; **37:**1399–1401.

30. Moreno-Jiménez B, Rodríguez-Carvajal R, Garrosa Hernández E, MoranteBenadero Ma. E: Terminal versus non-terminal care in physician burnout: the role of decision-making processes and attitudes to death. *Salud Mental* 2008; **31:**93–101.

31. Halbesleben JRB, Rathert C: Linking physician burnout and patient outcomes: exploring the dyadic relationship between physicians and patients. *Health Care Manage Rev* 2008; **33:**29–39.

32. Smets EMA, Visser MRM, Oort FJ, Schaufeli WB, de Haes H: Perceived inequity: does it explain burnout among medical specialists? *J Appl Soc Psychol* 2004; **34:**1900–1918.

33. McManus IC, Winder BC, Gordon D: The causal links between stress and burnout in a longitudinal study of UK doctors. *Lancet* 2002; **359:**2089–2090.

34. Deary IJ, Agius RM, Sadler A: Personality and stress in consultant psychiatrists. *Int J Soc Psychiatry* 1996; **42:**112–123.

35. Houkes I, Winants Y, Twellaar M: Specific determinants of burnout among male and female general practitioners: a cross-lagged panel analysis. *J Occup Organ Psych* 2008; **81:**249–276.

36. Goldberg R, Boss W, Chan L, Goldberg J, Mallon WK, Moradzadeh D, Goodman EA, McConkie ML: Burnout and its correlates in emergency physicians: four years' experience with a wellness booth. *Acad Emerg Med* 1996; **3:**1156–1164.

37. Leiter MP, Frank E, Matheson TJ: Demands, values, and burnout. *Can Fam Physician* 2009; **55:**1224–1225.

38. Travado L, Grassi L, Gil F, Ventura C, Martins C; Southern European Psycho-Oncology Study Group: Physician-patient communication among Southern European cancer physicians: the influence of psychosocial orientation and burnout. *Psychooncology* 2005; **14:**661–670.

39. Lee RT, Lovell BL, Brotheridge CM: Tenderness and steadiness: Relating job and interpersonal demands and resources with burnout and physical symptoms of stress in Canadian physicians. *J Appl Soc Psychol* 2010; **40:**2319–2342.

40. Truchot D: Career orientation and burnout in French general practitioners. *Psychol Rep* 2008; **103:**875–881.

41. Lisowska AGE: Professional burnout and stress among Polish physicians explained by the Hobfoll Resources Theory. *J Physiol Pharmacol* 2007; **58:**243–252.

42. Gabbe SG, Melville J, Mandel L, Walker E: Burnout in chairs of obstetrics and gynecology: diagnosis, treatment, and prevention. *Am J Obstet Gynecol* 2002; **186:**601–612.

43. Peisah C, Latif E, Wilhelm K, Williams B: Secrets to psychological success: Why older doctors might have lower psychological distress and burnout than younger doctors. *Aging Ment Health* 2009; **13:**300–307.

44. Surgenor LJ, Spearing RL, Horn J, Beautrais AL, Mulder RT, Chen P: Burnout in hospital-based medical consultants in the New Zealand public health system. *NZ Med J* 2009; **122:**11–18.

45. Ramirez AJ, Graham J, Richards MA, Cull A, Gregory WM: Satisfaction at work. *Lancet* 1996; **347:**724–728.

46. Lim RCH, Pinto C: Work stress, satisfaction, and burnout in New Zealand radiologists: comparison of public hospital and private practice in New Zealand. *J Med Imaging Radiat Oncol* 2009; **53:**194–199.

47. Barros D, Tironi M, Sobrinho C, Neves F, Bitencourt A, Almeida A, de Souza Y, Teles M, Feitosa A, Mota I, França J, Borges L, Lordão M, Trindade M, Almeida M, Filho E, José E, dos Reis: Intensive care unit physicians: socio-demographic profile, working conditions, and factors associated to the burnout syndrome. *Rev Bras Ter Intensiva* 2008; **20:**235–240.

48. Tokuda Y, Hayano K, Ozaki M, Bito S, Yanai H, Koiwmi S: The interrelationships between working conditions, job satisfaction, burnout and mental health among hospital physicians in Japan: a path analysis. *Ind Health* 2009; **47:**166–172.

49. Johns MM, Ossoff RH: Burnout in academic chairs of otolaryngology: head and neck surgery. *Laryngoscope* 2005; **115:**2056–2061.

50. Lert F, Chastang JF, Castano I: Psychological stress among hospital doctors caring for HIV patients in the late nineties. *AIDS Care* 2001; **13:**763–778.

51. Viviers S, Lachance L, Maranda M, Ménard C: Burnout, psychological distress, and overwork: the case of Quebec's ophthalmologists. *Can J Ophthalmol* 2008; **43:**535–546.

52. van der Ploeg E, Dorresteijn SM, Kleber RJ: Critical incidents and chronic stressors at work: their impact on forensic doctors.* J Occup Health Psychol* 2003; **8:**157–166.

53. McPhillips HA, Stanton B, Zuckerman B, Stapleton B: Role of a pediatric department chair: factors leading to satisfaction and burnout. *J Pediatr* 2007; **151:**425–430.

54. Ramirez AJ, Graham J, Richards MA, Cull A, Gregory WM, Learning MS, Snashall DC, Timothy AR: Burnout and psychiatric disorder among cancer clinicians. *Br J Cancer* 1995; **71:**1263–1269.

55. Kuhn G, Goldberg R, Compton S: Tolerance for uncertainty, burnout, and satisfaction with the career of emergency medicine. *Ann Emerg Med* 2009; **54:**106–113.

56. Arigoni F, Bovier PA, Mermillod B, Waltz P, Sappino AP: Prevalence of burnout among Swiss cancer clinicians, paediatricians and general practitioners: who are most at risk? *Support Care Cancer* 2009; **17:**75–81.

57. Bakker AB, Schaufeli WB, Sixma HJ, Bosveld W, Van Dierendonck D: Patient demands, lack of reciprocity, and burnout: longitudinal study among general practitioners. *J Organ Beh* 2000; **21:**425–441.

58. Krasner MS, Epstein RM, Beckman H, Suchman A, Chapman B, Mooney CJ, Quill TE: Association of an educational program in mindful communication with burnout, empathy, and attitudes among primary care physicians.*JAMA* 2009; **302:**1284–1293.

59. Fujimori M, Oba A, Koike M, Okamura M, Akizuki N, Kamiya M, Akechi T, Sakano Y, Uchitomi Y: Communication skills training for Japanese oncologists on how to break bad news.*J Cancer Educ* 2003; **18:**194–201.

60. Ratanawongsa N, Roter D, Beach MC, Laird SL, Larson SM, Carson KA, Cooper LA: Physician burnout and patient-physician communication during primary care encounters.*J Gen Intern Med* 2008; **23:**1581–1588.

61. Esquivel-Molina C, Buendia-Cano F, Martinez-Garcia O. Martinez-Mendoza J, Martinez-Ordaz V, Velasco-Rodríguez V: Burnout syndrome in medical staff affiliated to a tertiary care hospital (article in Spanish). *Rev Med Inst Mex Seguro Soc* 2007; **45:**427–436.

62. López-León E, Rodriguez-Moctezuma J, López-Carmona J, Peralta-Pedrero M, Munguía-Miranda C: Professional burnout in family physicians and its association with social demographic and labor factors (article in Spanish). *Rev Med Inst Mex Seguro Soc* 2007; **45:**13–19.

63. Escribà-Agüir V, Artazcoz L, Pérez-Hoyos S: Effect of psychosocial work environment and job satisfaction on burnout syndrome among specialist physicians (article in Spanish). *Gac Sanit* 2008; **22:**300–308.

64. Santos M, Abalo J: Burnout syndrome in medical staff working in neonatal intensive care units (article in Spanish). *Psicologia y Salud* 2005; **15:**25–32.

65. Lemaire JB, Wallace JE: Not all coping strategies are created equal: a mixed methods study exploring physicians' self reported coping strategies. *BMC Health Serv Res* 2010, **10:**208.

## Abbreviations

CI: Confidence interval; DP: Depersonalization; EE: Emotional exhaustion; HHR: Health human resources; OR: Odds ratio; PRISMA: Preferred reporting items for systematic reviews and meta-analyses; SD: Standard deviation; SE: Standard error; WHO: World Health Organization.

## Competing interests

The authors declare that they have no competing interests.

## Authors**’** contributions

RL designed the study, interpreted data, wrote first and revised drafts, and constructed figures. BS analyzed and interpreted data, constructed tables, and critically evaluated both drafts for content. SH compiled and analyzed data, and critically evaluated both drafts for content. BL collected, compiled and interpreted data, created the categories for study variables, and participated in writing both drafts. LS collected, compiled and analyzed data, critically evaluated first draft for content, and participated in writing the revised draft. All authors read and approved the final manuscript.
